# Quantitative identification of dynamical transitions in a semiconductor laser with optical feedback

**DOI:** 10.1038/srep37510

**Published:** 2016-11-18

**Authors:** C. Quintero-Quiroz, J. Tiana-Alsina, J. Romà, M. C. Torrent, C. Masoller

**Affiliations:** 1 Universitat Politècnica de Catalunya, Departament de Física, Colom 11, 08222 Terrassa, Barcelona, Spain

## Abstract

Identifying transitions to complex dynamical regimes is a fundamental open problem with many practical applications. Semi- conductor lasers with optical feedback are excellent testbeds for studying such transitions, as they can generate a rich variety of output signals. Here we apply three analysis tools to quantify various aspects of the dynamical transitions that occur as the laser pump current increases. These tools allow to quantitatively detect the onset of two different regimes, low-frequency fluctuations and coherence collapse, and can be used for identifying the operating conditions that result in specific dynamical properties of the laser output. These tools can also be valuable for analyzing regime transitions in other complex systems.

Complex systems often undergo abrupt or gradual transitions to dynamical regimes that can be safe or dangerous for the system functionality^1^. Examples of dangerous transitions include desertification, population extinctions, financial crashes, cardiac arrhythmia, epileptic seizures, etc.^2^. A precise identification of such transitions is important for preventing harmful consequences, and a lot of efforts have focused on developing reliable diagnostic tools that can be applied to observed time-series which are finite and usually stochastic^3^,^4^,^5^,^6^.

Semiconductor lasers with optical feedback can generate a rich variety of dynamical behaviors^7^, and thus, are ideal testbeds for studying dynamical transitions and testing novel analysis tools^8^. Semiconductor lasers with optical feedback are also important practical devices, because the complex output signals that they generate can be exploited for several applications^9^,^10^, including sensors^11^,^12^, ultra-fast random number generation^13^,^14^, reservoir computing^15^,^16^,^17^, etc.

Two well-known dynamical regimes are the low-frequency fluctuations (LFFs) and the coherence collapse (CC). The LFF regime is characterized by sudden dropouts of the laser intensity, followed by gradual, step-like recoveries. In contrast, the CC regime, which occurs at higher pump currents, is characterized by fast and irregular intensity fluctuations. Both regimes occur with moderated feedback levels and when the feedback delay time is much longer than the relaxations oscillation period of the laser. The intensity dropouts are actually a slow modulation of a series of fast, picoseconds pulses^18^, which are well modeled by the Lang-Kobayashi (LK) equations^19^.

The LFFs and CC regimes have been known for decades and their dynamical origin and statistical properties have been intensively studied^20^,^21^,^22^,^23^,^24^,^25^,^26^,^27^,^28^,^29^,^30^,^31^,^32^,^33^,^34^,^35^,^36^,^37^,^38^,^39^,^40^,^41^,^42^,^43^,^44^,^45^,^46^,^47^,^48^,^49^,^50^,^51^,^52^,^53^,^54^,^55^,^56^,^57^,^58^. However, to the best of our knowledge, the transition points from noisy emission to LFFs, and from LFFs to CC, occurring as the pump current increases, have not yet been quantified. As can be seen in the video included in Supplementary Material, in spite of the fact that the dynamical regimes are profoundly different, the transitions are gradual and an objective identification of the transition points is not possible by a simple inspection of the time series.

Here we address the following questions: can these regimes be quantitatively distinguished? can the onset of each regime be quantitatively identified? We show that, by using three diagnostic tools applied to experimental intensity time-series, are able to quantify these transitions. We use these tools to analyze how noisy fluctuations (close to threshold) gradually transform into well-defined dropouts (at higher pump currents), which then merge into fast and irregular fluctuations (at even higher pump currents). We delimit the coexistence region, where the dropouts alternate with stable noisy emission^59^,^60^,^61^ and find a region of pump currents where occasionally, extremely depth dropouts occur. In the SI we demonstrate the robustness of our results by presenting a second set of experiments, and interpret our findings in terms of simulations of the LK model.

## Results

**Figure 1:**
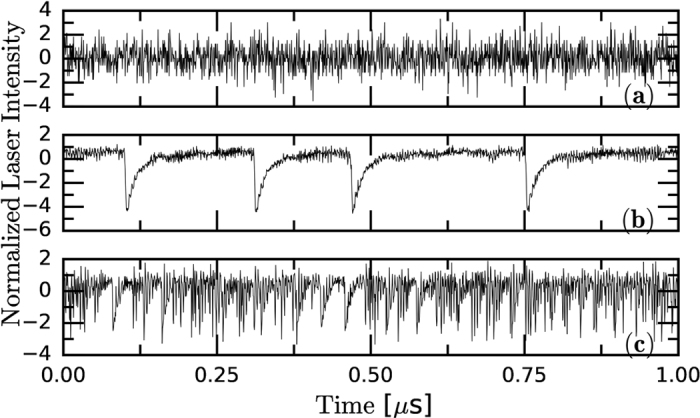
Typical intensity time-series, normalized to zero mean and unit variance: (**a**) noisy fluctuations, (**b**) dropouts in the LFF regime and (**c**) fast fluctuations in the CC regime. The laser pump current, normalized to the threshold current of the solitary laser is I/I_th_ = 0.95, 1.02 and 1.20, respectively. The horizontal axis is the same in the three panels.

**Figure 2:**
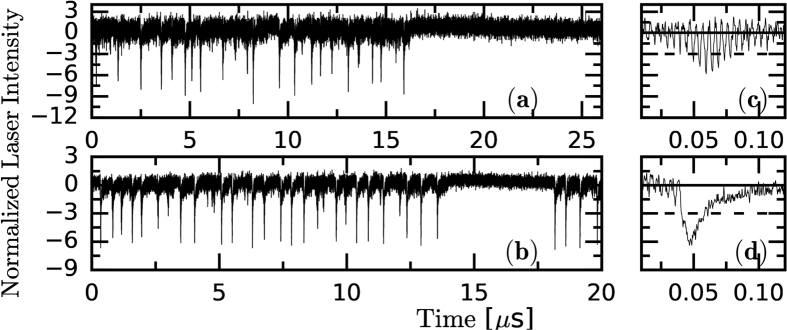
Intensity time-series (normalized to zero mean and unit variance) for I/I_th_ = 0.97 (**a**) and 0.98 (**b**). Note the different time-scale with respect to Fig. 1. In panel (**a**) the depths of the dropouts are heterogeneous and there are dropouts below −9*σ*. In panel (**b**) the dropouts are of similar depth and they are less pronounced. The panels (**c**) and (**d**) display a detail of a single dropout: it is abrupt in (**d**), while is more gradual in (**c**). The horizontal dashed and solid lines in panels (**c**) and (**d**) stand for the 3*σ* level and the average value respectively.

The experiments were carried out with a semiconductor laser with optical feedback as in ref. 62 (see *Methods*). In Fig. 1 typical intensity time-series are depicted corresponding to the regimes of noisy fluctuations, LFFs, and CC. Recording the intensity dynamics over longer intervals allows to study the alternation of noisy fluctuations and LFFs, shown in Fig. 2. The detection system uses an amplifier that removes the mean value of the signal, and thus, the zero intensity level is equal to the mean value of the intensity waveform. To quantitatively characterize, as the pump current increases, the transitions between these dynamical regimes, we use three diagnostic tools that capture different properties of the intensity time-series.

### First diagnostic tool

**Figure 3:**
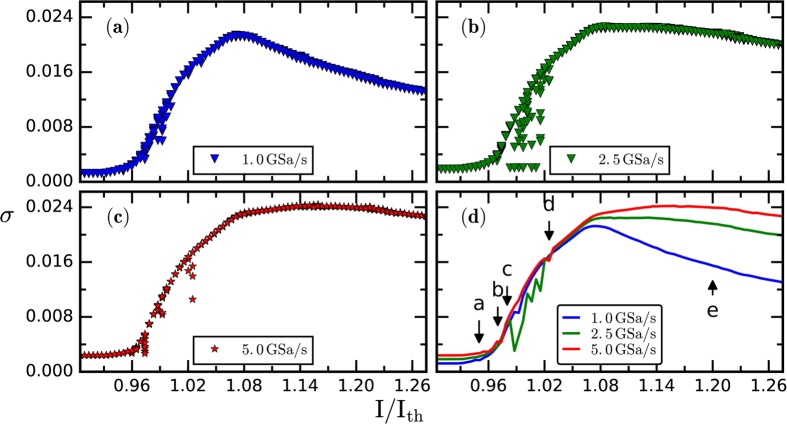
Standard deviation of the intensity time series, *σ*, recorded using three different sampling rates, vs the laser pump current, normalized to the threshold value, I/I_th_. In panels (**a**–**c**), for each pump current and sampling rate, ten *σ* values are shown; in panel (**d**), the average *σ* value is plotted vs the normalized laser pump current, for the three sampling rates. In this panel the arrows indicate the current values where the behaviors discussed in the text occur.

The first method is based in the analysis of the standard deviation, *σ*, of intensity time-series recorded with different oscilloscope sampling rate. Figure 3 displays *σ* vs. the laser pump current, for three sampling rates. In panels (a)–(c), for each pump current, ten *σ* values are displayed, computed from ten time series recorded under identical conditions; in panel (d), for each set (pump current, sampling rate), the mean *σ* value is displayed, and in this plot we can identify five behaviors as the pump current increases:

- Close to the lasing threshold *σ* is small and shows a low variability. This corresponds to stable noisy emission, shown in Fig. 1(a).
- For higher current *σ* increases gradually and shows higher variability, capturing the development of intensity dropouts (i.e., the onset of the LFF regime). A typical intensity time-trace is shown in Fig. 2(a).
- For slightly higher current there is a wide spread in the values of *σ*. This captures the coexistence between stable noisy emission and well-defined LFF dropouts^59^,^60^,^61^. A typical intensity time-trace is shown in Fig. 2(b).
- For higher currents there is an almost linear increase of *σ*, which captures the increase of the depth and of the frequency of the dropouts. A typical intensity time-trace is shown in Fig. 1(b). A similar linear grow was reported in ref. 42.
- Finally, for pump currents above I/I_th_ ~ 1.08, *σ* saturates or decreases, depending on the sampling frequency. This change, previously unrecognized, captures the fact that the dropouts become irregular and quantitatively identifies the onset of coherence collapse. A typical intensity time-trace is shown in Fig. 1(c).

### Second diagnostic tool

The second method is based in the analysis of the number of intensity dropouts. A dropout is detected each time the intensity decreases below a preselected threshold (in the following, referred to as *detection threshold*). Because the depth of the dropouts depends on the pump current, in order to be able to use a criterion to define a dropout that holds for all pump currents, each intensity time series is normalized to unit variance. Then, detection thresholds lower than −1 are used to detect ‘dropout-like’ events. To avoid detecting as events the fluctuations that occur during the recovery process (after a dropout), a second threshold is used: the intensity has to grow above the mean value (which is zero due to the amplifier used in the setup), before another event can be detected. We use a sampling frequency of 5 GSa/s because it provides a good compromise between a precise detection of the individual threshold-crossing events, and detecting a large number of events.

**Figure 4:**
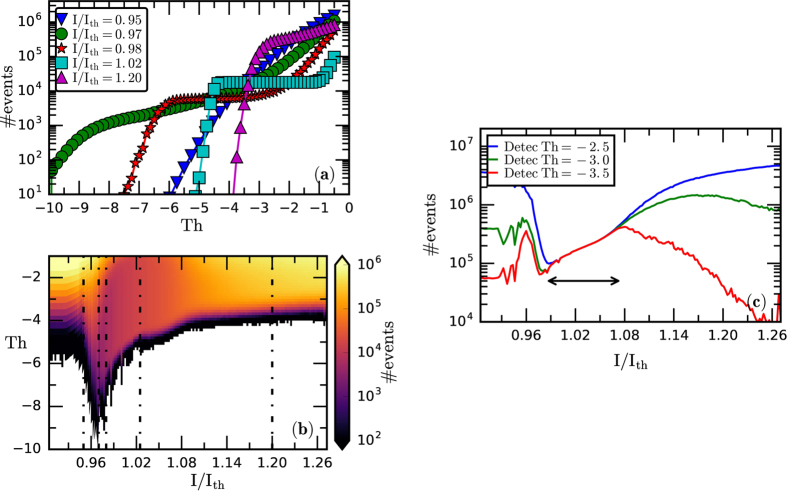
(**a**) Number of events (in logarithmic scale) as a function of the detection threshold (in units of *σ*), for different pump currents. (**b**) Number of events in color code (logarithmic scale) vs. the pump current and the detection threshold. The white color indicates that no events are detected. (**c**) Number of events in color code (logarithmic scale) vs. the pump current for three detection thresholds. The arrow indicates the boundaries of the LFF region, where the depth of the intensity dropouts is regular and thus, the number of events is the same for the three thresholds.

In Fig. 4(a) the number of events (averaged over ten time-series, in logarithmic scale) is plotted vs. the detection threshold, for different pump currents, which correspond to the different behaviors identified in the previous analysis of *σ* (the corresponding intensity probability distribution functions are shown in the SI):

- At low pump current [inverted triangles, the time-series was shown in Fig. 1(a)] the number of events decreases smoothly with the threshold, which is consistent with Gaussian statistics.
- At higher pump current [circles, the time-series was shown in Fig. 2(a)] the number of events gradually decreases with the detection threshold, capturing the fact that the intensity distribution develops a tail, due to the dropouts. While there are about 10^6^ events deeper than −1, few are deeper than −9 (~100).
- At slightly higher pump current [stars, the time-series was shown in Fig. 2(b)] a plateau develops, which indicates that there is a range of thresholds for which the number of events is robust with respect to the threshold (thresholds in between −6 and −3 detect about 10^4^ events). This plateau captures the fact that many dropouts are of similar depth. We note that the dropouts are less pronounced than those occurring at slightly lower pump current, because no event crosses the −8 threshold.
- At higher pump current [squares, the time-series was shown in Fig. 1(b)] the plateau occurs in between −5 and −1 (thresholds in this range detect more than 10^4^ events), capturing the fact that the dropouts become more frequent and less depth in units of *σ*.
- For the higher pump current [triangles, the time-series was shown in Fig. 1(c)] the plateau disappears and the number of events decreases sharply with the threshold, which indicates non-Gaussian statistics.

These findings are summarized in Fig. 4(b) that displays the number of events (in logarithmic color code) vs. the pump current and the detection threshold. The plots shown in Fig. 4(a) are obtained by moving along the dashed vertical lines in Fig. 4(b). We note that at low pump current there are no events below −6 threshold (the white color indicates that no threshold-crossings are detected), but as the pump current increases, the detection threshold ‘grows’ (negatively), due to the fact that dropouts gradually emerge. Then, we observe a narrow region of pump currents, 0.96 < I/I_th_ < 0.99, where very few events (~100) are detected with thresholds below −8. Thus, this allows delimiting the pump current region where extremely depth dropouts occur. A further increase of the pump current results in a gradual increase of the detection threshold that captures the fact that the dropouts become less pronounced. We also note that for pump currents above I/I_th_ ~ 1.08 the number of events increases (note the change from dark to lighter color). This captures the fact that the dropouts occur more often, and quantitatively identifies the onset of coherence collapse, in good agreement with the analysis of *σ*. The transitions can also be observed when plotting the number of events vs. the pump current, for different detection thresholds. As shown in Fig. 4(c), there is a well-defined region where the number of detected events is the same for the different thresholds considered. This reveals that in this region the depth of the intensity dropouts is regular, and thus, quantitatively identifies the boundaries of the LFF region. In contrast, outside this region the number of detected events varies with the threshold, capturing the fact that the depth of the intensity dropouts is irregular.

### Third diagnostic tool

The third method is based in the analysis of the time intervals between consecutive threshold crossings (inter-event-intervals, IEIs). We use a symbolic method of time-series analysis known as *ordinal analysis*^63^, which has proven valuable for studying laser nonlinear dynamics^47^,^48^,^49^,^52^,^53^,^54^,^62^. With this method, each sequence of IEIs is transformed into a sequence of ordinal patterns (OPs), defined by considering the relative length of *D* consecutive IEIs. For example, if *D* = 2 there are two OPs: Δ*T*_*i*_ < Δ*T*_*i*+1_ gives ‘01’ and Δ*T*_*i*_ > Δ*T*_*i*+1_ gives ‘10’; for *D* = 3 there are six OPs: Δ*T*_*i*_ < Δ*T*_*i*+1_ < Δ*T*_*i*+2_ gives ‘012’, Δ*T*_*i*+2_ < Δ*T*_*i*+1_ < Δ*T*_*i*_ gives ‘210’, etc.

The different dynamical regimes and transitions are then characterized in terms of the probabilities of occurrence of the OPs in the IEI sequence. This allows detecting temporal correlations in the sequence of events: if the OPs are equally probable there is no temporal structure in the IEI sequence, while more probable and/or less probable OPs reveal the presence of temporal ordering.

To detect the events we first consider a fixed threshold, equal to −3 because it provides a good compromise between analyzing only the dropouts that are sufficiently depth (filtering noisy fluctuations), while keeping a large number of dropouts (needed to compute OP probabilities with good accuracy), in a wide range of pump currents. As shown in Fig. 4(b), the detection threshold varies in a nontrivial way with the pump current. With −3, more than 75000 events are detected, for all pump currents.

**Figure 5:**
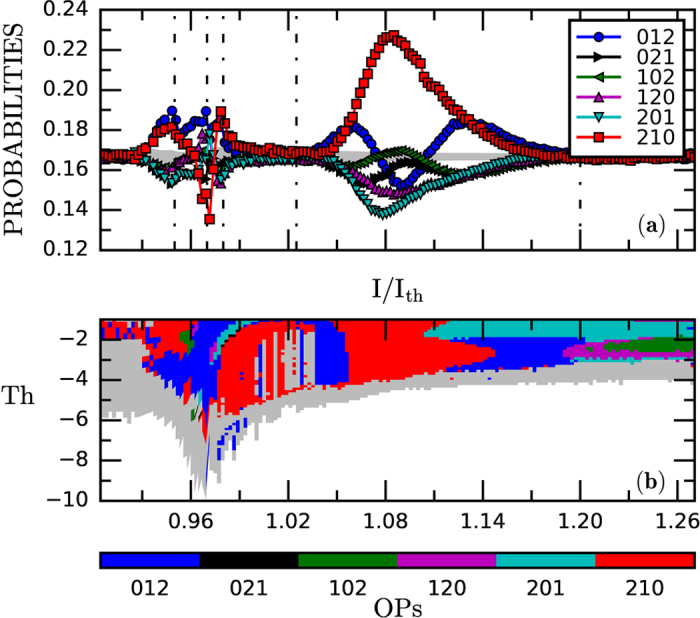
(**a**) Probabilities of the six *D* = 3 ordinal patterns vs. the normalized pump current, I/I_th_. The gray region indicates the range of probability values that is consistent with the uniform distribution, which is estimated with a binomial test: considering a confidence level of 95%, if all the OP probabilities are within the range, *p* ± 3*σ*_*p*_, where *p* = 1/6 and 
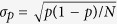
 (with *N* being the length of the dataset), the OP are equally probable; in contrast, if at least one probability value is above *p* + 3*σ*_*p*_ or below *p* − 3*σ*_*p*_, the OPs are not equally probable, with 95% confidence level. (**b**) Most probable OP [in the same color code as panel (a)] vs. the normalized pump current and the detection threshold. In the gray regions, either the six OPs are equally probable, or the number of detected events is not enough to compute the OP probabilities with robust statistics; the white color indicates that no events are detected.

Figure 5(a) displays the six OP probabilities vs. the pump current. At low pump current the OPs are equally probably, which is consistent with uncorrelated intensity fluctuations. At higher pump currents, large and abrupt variations of the OP probabilities are seen. This is the pump current region where the dropouts develop, they are heterogeneous and few of them are very depth. The OP probabilities uncover temporal correlations which are due to the fact that, in this current region, the −3 threshold detects events during the recovery process. It is worthwhile to note that the shape of the dropout waveform changes in this current region [see the panels (c) and (d) in Fig. 2], and the OP probabilities capture this change.

At higher pump currents the OP probabilities vary smoothly and pattern ‘210’ becomes the most probable pattern. We note that the value of the pump current at which the probability of pattern ‘210’ is maximum, I/I_th_ = 1.08, is also the one where the onset of coherence collapse occurs, as identified by the other two diagnostic tools. For higher pump currents the OP probabilities detect additional changes in the temporal correlations among consecutive events, as pattern ‘012’ becomes the most probable one, and then, for even higher pump currents, all patterns become about equally probable (consistent with no temporal correlations among consecutive events).

Next, we analyze the influence of the detection threshold. Figure 5(b) displays in color code the most probable OP vs. the pump current and the detection threshold. It can be observed that there is a range of pump currents where the most probable pattern does not vary with the detection threshold (0.96 < I/I_th_ < 1.11). In this region the depth of the dropouts is regular; in contrast, for other pump currents the most probable OP either varies with the detection threshold (because the depth of the dropouts is irregular), or it is not defined (because the OP probabilities are very similar).

**Figure 6:**
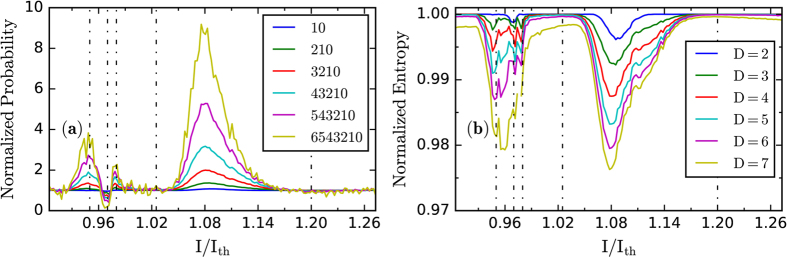
(**a**) Probability of the “decreasing trend” pattern of length *D* with *D* = 2 … 7 (i.e., patterns 10, 210, 3210, …, 6543210) vs. the normalized pump current, I/I_th_. For easy comparison, each probability is normalized to the value expected if the patterns are equally probable, 1/*D*!. (**b**) Normalised permutation entropy computed from the probabilities of the patterns of length *D* (with *D* = 2 … 7) vs. the normalized pump current. In both panels (**a**) and (**b**) regime transition points are clearly identified, which are consistent with the transition points that were detected in Fig. 5 with *D* = 3.

Lastly, we analyze the influence of the length of the ordinal pattern, *D*. Figure 6(a) displays the probability of the “decreasing trend” pattern of length *D* with *D* = 2 … 7 (i.e., the probability of pattern 10, 210, 3210, …, 6543210) vs. the normalized pump current. For easy comparison, each probability is normalized to the value expected if the patterns are equally probable, 1/*D*!. In this plot, the regime transition points that were identified with *D* = 3 in Fig. 5 are also observed for the other values of *D* considered. Moreover, the entropy of the probabilities of the ordinal patterns (known as permutation entropy^63^,^64^), normalised to its maximum value, is another diagnostic tool that also allows to identify the regime transition points, as shown in Fig. 6(b).

## Discussion

To summarize, we have used three analysis tools to identify and characterize transitions between different dynamical regimes displayed by semiconductor lasers with optical feedback, as the laser pump current increases. These tools capture different properties of these regimes and *quantitatively* distinguish among stable noisy emission, coexistence between stable noisy emission and low-frequency fluctuations (LFFs), LFFs, and coherence collapse (CC).

Previous statistical studies have focused on the properties of the intensity distribution or on the timing of the intensity dropouts. While the statistical tools we have used are standard, taken together they have allowed us to simultaneously quantify the properties of both, the timing and the depth of the dropouts. A main conclusion of our analysis is that the change in the shape of the curve of the standard deviation vs. the pump current (shown in Fig. 3), which is accompanied by a maximum in the probability of pattern 210 (shown in Fig. 5 and occurring at the same value of the laser pump current), quantitatively determine the transition from LFFs to CC regime. We also found that at the onset of LFFs, rare and extremely depth dropouts occur. These analysis tools also provide objective measures for delimiting the borders of the pump current region where stable emission and the LFF regime coexist. In the Supplementary Information we demonstrate the robustness of these observations with experiments performed with a different laser and feedback conditions, and we also provide an interpretation of our findings in terms of simulations of the LK model.

These analysis tools can be used for identifying the optimal operating conditions that result in specific properties of the laser intensity, and thus, we expect that they will be valuable for applications that exploit the complex output signals generated by semiconductor lasers with optical feedback. These tools can also be valuable for characterizing transitions in other complex systems.

## Methods

In the experiments we used a 658 nm AlGaInP semiconductor laser (Hitachi HL6501MG, threshold current I_th,sol_ = 43.14 mA) with optical feedback as in ref. 62. The feedback-induced threshold reduction and the feedback delay time are 7% and 4.7 ns respectively. The laser temperature and current were stabilized using a combi-controller (Thorlabs ITC501) with an accuracy of 0.01 C and 0.01 mA, respectively. During the experiments the temperature was set to *T* = 18 C. The output intensity was detected with a photo detector (Thorlabs DET210) connected to an amplifier (FEMTO HSA-Y-2-40) and recorded with an oscilloscope (Agilent Infiniium DSO9104A) using different sampling frequencies. A LabVIEW program was used to control the experiment. For each set of (pump current, sampling frequency), 10 time series with 10^7^ intensity data points each were recorded.

## Additional Information

**How to cite this article**: Quintero-Quiroz, C. *et al*. Quantitative identification of dynamical transitions in a semiconductor laser with optical feedback. *Sci. Rep.* **6**, 37510; doi: 10.1038/srep37510 (2016).

**Publisher’s note:** Springer Nature remains neutral with regard to jurisdictional claims in published maps and institutional affiliations.

## Supplementary Material

Supplementary Information

Supplementary Information
