# Supplementary material for: Quantitative identification of dynamical transitions in a semiconductor laser with optical feedback
Source: Sci Rep. 2016 Nov 18;6:37510. doi: 10.1038/srep37510 (PMC5114591; doi:10.1038/srep37510)
Supplement: Supplementary Information [file srep37510-s1.pdf]

Supplementary Information for "Quantitative identification of dynamical transitions in a semiconductor laser with optical feedback"

C. Quintero-Quiroz, J. Tiana-Alsina, Jordi Romà, M. C. Torrent, C. Masoller
Departament de Física, Universitat Politècnica de Catalunya, Colom 11, 08222 Terrassa, Barcelona, Spain

October 25, 2016

Supplementary Information for “Quantitative identification of dynamical transitions in a semiconductor laser with optical feedback”

C. Quintero-Quiroz, J. Tiana-Alsina, Jordi Romà, M. C. Torrent, C. Masoller
Departament de Física, Universitat Politècnica de Catalunya, Colom 11, 08222 Terrassa, Barcelona, Spain

Here we include multimedia information: we present a video of the experimental dynamical transitions studied in the main text. We also complement the analysis done in the main text by presenting the characterization of the laser output intensity using standard techniques: the light power vs. current characteristic (LI curve) and the probability density function (pdf) of the laser intensity. We also demonstrate the robustness of our findings by presenting a second set of experiments performed with a different laser, under different feedback conditions. In addition, we present simulations of the Lang-Kobayashi model, which show good qualitative agreement with the observations.

Video

In the video included as supplementary material we observe, as the pump current is ramped, the gradual transitions studied in the main text: from noise intensity fluctuations, to a regime where occasionally rare intensity dropouts occur, which then become more regular and frequent in the low frequency fluctuations region, and finally, with further increase of the laser current, the regular and well-defined intensity dropouts transform into fast and highly irregular intensity fluctuations.

LI curve and intensity pdf

Figure 1 displays the light power vs. current characteristic (LI curve), and we indicate the pump current values where the different regimes discussed in the main text occur. For easy comparison, we also show the standard deviation of intensity fluctuations.

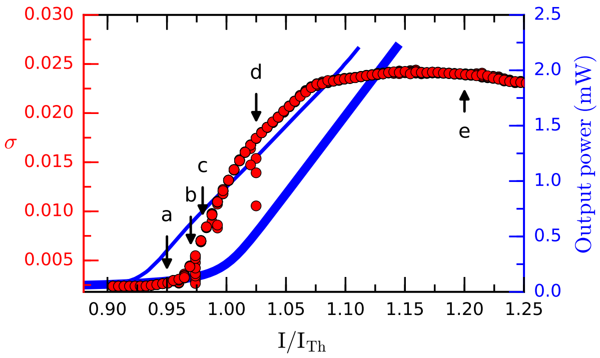

Figure 1: Output power (vertical right axis) and the standard deviation of the intensity fluctuations (vertical left axis) vs. the pump current, normalized to the solitary laser threshold current. The thin and thick lines represent the output power with and without optical feedback respectively. The letters indicate the values of the pump current the where different behaviors discussed in the text occur.

As discussed in the main text, in the LFF regime, due to intensity dropouts, the probability density function (pdf) of the intensity fluctuations, shown in Fig. 2, develops a tail in the

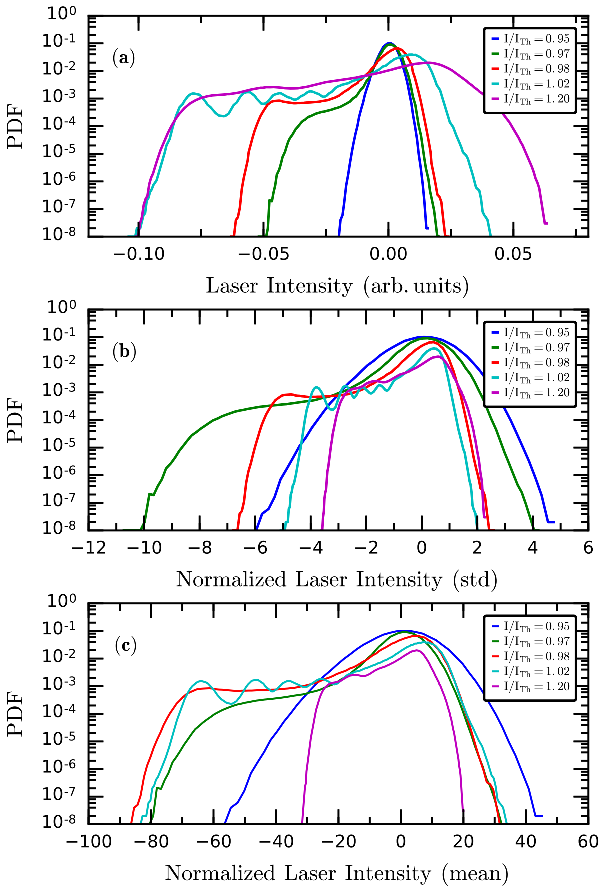

Figure 2: Probability density function (pdf) of the laser output intensity for five values of the normalized pump current, corresponding to the different dynamical regimes discussed in the main text. For easy comparison, in panel (a) the intensity is displayed in arbitrary units (raw data recorded by the oscilloscope), in panel (b), it is normalized to the standard deviation, and in panel (c), to the average output power (as shown in Fig.1).

left side of the distribution. In contrast, before the onset of the LFF regime ($I/I_{th} = 0.95$), and

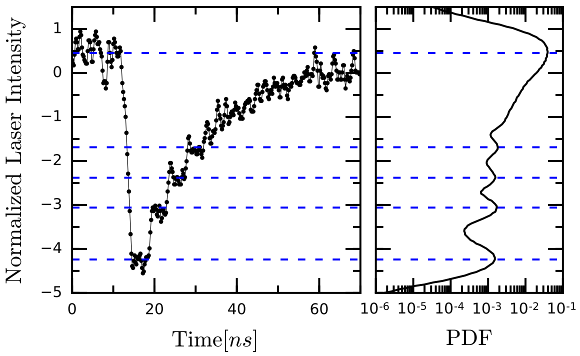

Figure 3: Detail of an intensity dropout when the laser pump current, normalized to the solitary threshold, is $I/I_{th} = 1.02$.

in the CC regime ($I/I_{th} = 1.2$), the intensity pdf has a well defined cutoff. While at low current the pdf is Gaussian, in the CC regime the pdf is not Gaussian. We note that for $I/I_{th} = 1.02$ the pdf displays a nontrivial structure which is due to the step-like recovery that occurs after a dropout, as shown in Fig. 3. These observations are in agreement and consistent with previous findings [1, 2, 3, 4, 5].

Second set of experimental observations

Here we present experiments performed with a different laser and feedback conditions compared to those in the main text, and we find qualitatively very similar results. The laser is a 685 nm HL6750MG semiconductor laser (Opnext HL6750MG) with solitary threshold current of $I_{th} = 28.29$ mA. The feedback-induced threshold reduction and the feedback delay time are 15.42% and 5.3 ns respectively.

Figure 4 displays the standard deviation, σ, of the intensity time-series vs. the laser pump

current, for a sampling frequency of 5 GSa/s of the oscilloscope, and a very good agreement is seen with Fig. 3 in the main text. Figure 5(a) displays the number of events vs. the detection threshold, and here again a qualitative good agreement is found with Fig. 4(a) of the main text. It is worthwhile to note that the plateau also exists with a different detection method, as shows Fig. 5(c): instead of normalizing the time series to standard deviation equal to one, we normalize such that the maximum and minimum are equal to one and zero respectively. We note that these two methods differ in the sense that with the second method any threshold value within (0,1) will detect a certain number of events, while with the first method (used in the main text), the interval of detection thresholds depends on the pump current [as shown in Fig. 5(b) and Fig. 4(b) of the main text]. Nevertheless, with this alternative normalization one can also observe the existence of the plateau.

Figure 6 displays the six OP probabilities vs. the pump current. We note a variation very similar to that shown in Fig. 5(a) in the main text.

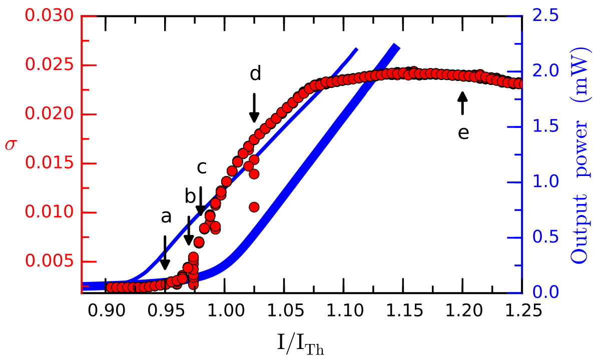

Figure 4: Second set of experimental results: standard deviation of intensity time series, σ, vs the normalized pump current, I/I_{th}. This plot is very similar to Fig. 3 in the main text.

MODEL

In order to further demonstrate the robustness of the experimental findings presented in the main text, we performed simulations of the Lang and Kobayashi (LK) rate equations [6] for the slowly varying complex electric field E and the carrier density N. The model equations are:

$$\frac{dE}{dt} = \frac{1}{2\tau_p}(1+\alpha)(G-1)E + \eta E(t-\tau)e^{-i\omega_0\tau} + \sqrt{2\beta_{sp}}\,\xi \quad (1)$$

$$\frac{dN}{dt} = \frac{1}{\tau_N}\left(\mu - N - G\,|E|^2\right) \quad (2)$$

where α is the linewidth enhancement factor, τ_p and τ_N are the photon and carrier lifetimes respectively, $G = N/(1+\varepsilon\,|E|^2)$ is the optical gain (with ε a saturation coefficient), μ is the pump current parameter (which is equal to the experimental control parameter –the normalized pump current– only at the solitary threshold [12], where both are equal to 1), η is the feedback coupling coefficient, τ is the feedback delay time, ω_0 is the solitary laser frequency, $\omega_0\tau$ is the feedback phase, β_{sp} is the noise strength, representing spontaneous emission, and ξ is a Gaussian distribution with zero mean and unit variance. The model equations were simulated with typical parameters as in [11] [$\tau_p = 0.00167$ ns, $\tau_N = 1$ ns, $\alpha = 4.0$, $\varepsilon = 0.01$, $\eta = 10$ ns^{-1}, and $\tau = 5$ ns, $\beta_{sp} = 5\times10^{-5}ns^{-1}$].

Numerical results

In the framework of the LK model, it has been shown that the LFF intensity dropouts can be either transient or sustained [7, 8], with the probability of observing sustained LFFs or stable emission depending on the relative widths of the win-

dows where these regimes occur. For typical parameters, however, the LFF are a transient dynamics with a duration that increases with the pump current parameter [9, 10]. Typical intensity time-series are shown in Fig. 7.

To compare with experimental observations we need to generate a sufficiently large number of dropouts, therefore, for each value of the pump current parameter, 20 trajectories of 50 μs were generated from random initial conditions.

In Fig. 8 we show that, taken together, the results of the analysis of the simulated data are in very good qualitative agreement with the experimental observations: the variation of the standard deviation, Fig. 8(a), the variation of the number of threshold-crossing, Fig. 8(b), and the variation of the OP probabilities, Fig. 8(c), with the pump current parameter are very similar to those encountered in the experimental data. The comparison between the shape of the experimental and simulated σ curve, shown in Fig. 9, allowed us to determine the five values of the pump current parameter that correspond to the experimental pump currents analyzed in the main text. For those values, as shown in Fig. 8(b), the variation of the number of events is very similar to that seen in the experiments.

However, it is worthwhile to note that the agreement is only qualitative: we note that the simulated dropouts are less depth than the experimental ones [in Fig. 8(b) the lowest detection threshold is -4σ]. A second discrepancy is seen in Fig. 9, where the experimental and simulated σ curves agree qualitatively well only if the horizontal axes are shifted (i.e., $\mu = 1$ is shifted with respect to $I/I_{th} = 1$) and the vertical axes are re-scaled. The origin of these discrepancies could be the fact that in the simulations the LFFs are transient; also, the simple filtering used (a moving average in a time-window of 5 ns) might play a role. We remark that our goal here is only to demonstrate the robustness of our findings though a comparison with model simulations.

To conclude this comparison, in Fig. 10 we present the equivalent of Fig. 1, computed from the simulated time-series. Here again we observe a good qualitative agreement model simulations – experimental observations.

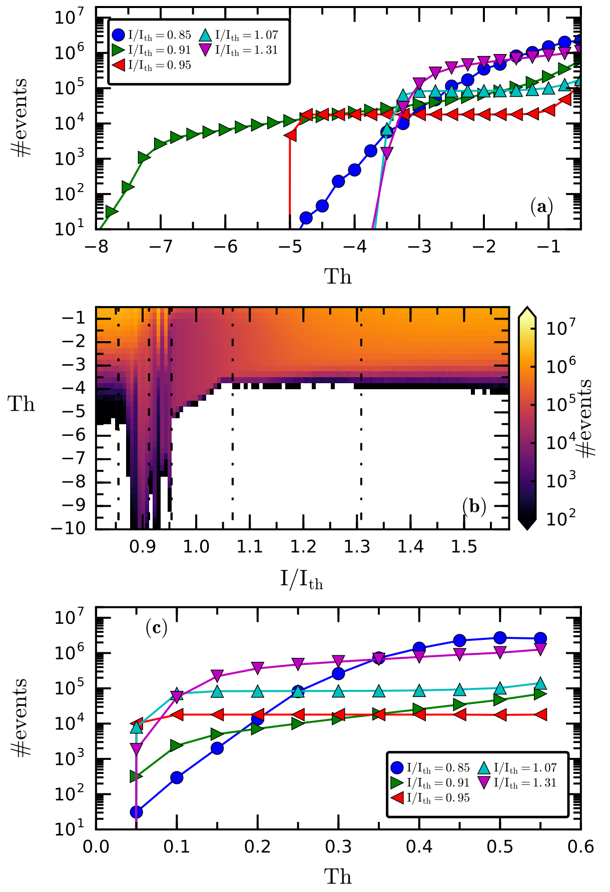

Figure 5: Second set of experimental results: (a) Number of events (in logarithmic scale) as a function of the detection threshold (in units of the standard deviation of the intensity fluctuations), for five values of the normalized pump current. A qualitative good agreement is seen with Fig. 4(a) of the main text. (b) Number of events in color code (logarithmic scale) vs. the pump current and the detection threshold. (c) Number of events (in logarithmic scale) as a function of the detection threshold (in this case the time series was normalize such that the maximum and minimum are equal to one and zero respectively), for five values of the normalized pump current.

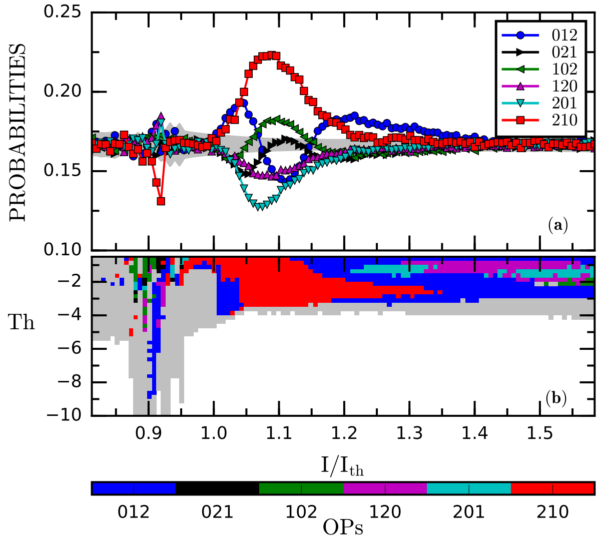

Figure 6: Second set of experimental results:(a) Probabilities of the six $D = 3$ ordinal patterns vs. the normalized pump current, $\mathrm{I/I_{th}}$. A qualitative good agreement with Fig. 5(a) (in the main text) can be observed. (b) Most probable OP vs. the normalized pump current and the detection threshold.

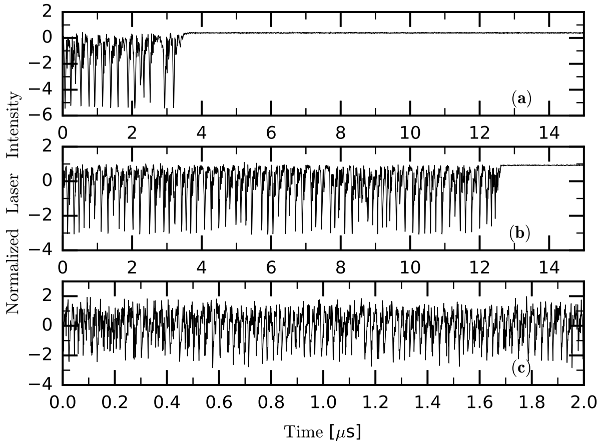

Figure 7: Simulated time series for three values of the pump current parameter, $\mu = 0.978$ (a), 0.982 (b) and 1.032 (c)

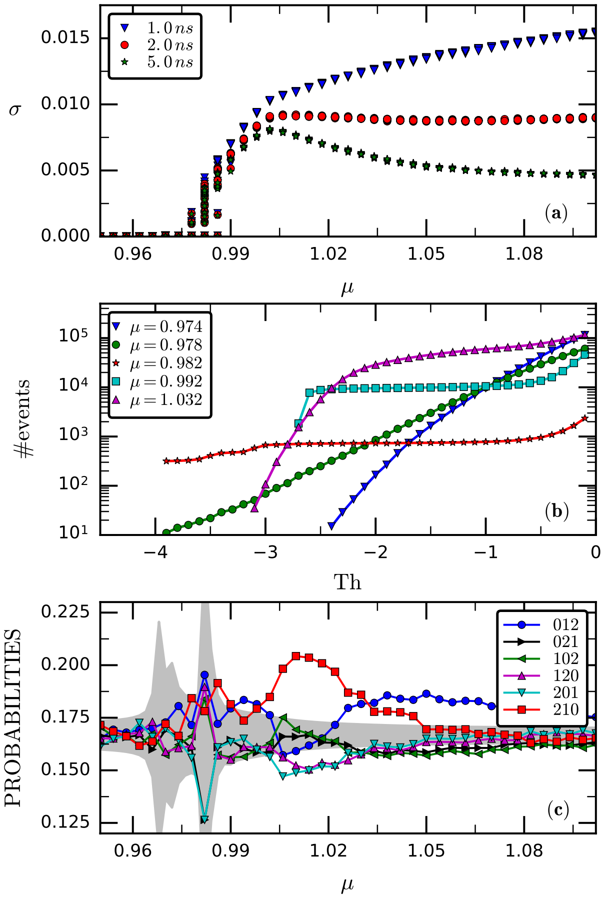

Figure 8: Results of numerical simulations. (a) Standard deviation of intensity time series, σ, vs the pump current parameter, μ. (b) Number of events (in logarithmic scale) as a function of the detection threshold. (c) Probabilities of the six $D = 3$ ordinal patterns vs. μ.

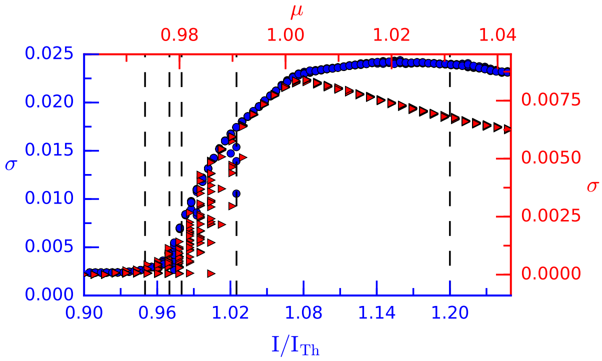

Figure 9: In the vertical left and bottom horizontal axis (in blue): standard deviation of the experimental intensity time series, σ, vs. the normalized pump current $\mathrm{I/I_{th}}$; in the vertical right and upper horizontal axis (in red): standard deviation of the simulated intensity time series vs. the pump current parameter, μ.

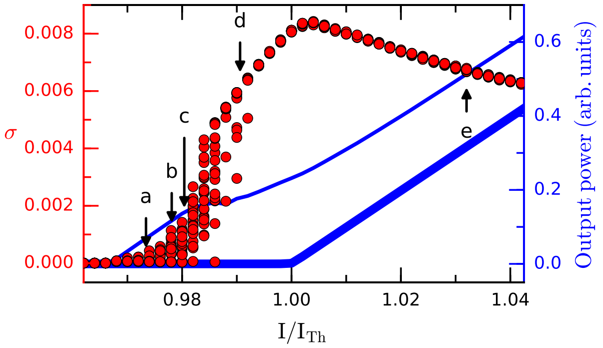

Figure 10: As Fig. 1 but computed from simulated data.

Bibliography

[1] Y. Liu, P. Davis, and Y. Takiguchi, Phys. Rev. E. **60**, 6595 (1999).

[2] G. Huyet et al., Phys. Rev. A. **60**, 1534 (1999).

[3] N. Q. Li et al., Opt. Lett. **39**, 5949 (2014).

[4] N. Q. Li et al., IEEE J. Sel. Top. Quantum Electron. **21**, 1 (2015).

[5] D. Choi et al., Phys. Rev. E **93**, 042216 (2016).

[6] R. Lang and K. Kobayashi, IEEE J. Quantum Electron. 16, 347 (1980).

[7] R. L. Davidchack et al, Phys. Lett. A. 267, 350 (2000).

[8] R. L. Davidchack et al, Physica D 145, 130 (2000).

[9] A. Torcini, S. Barland, G. Giacomelli, and F. Marin, Phys. Rev. A **74**, 063801 (2006).

[10] J. Zamora-Munt, C. Masoller, and J. García-Ojalvo, Phys. Rev. A **81**, 033820 (2010).

[11] T. Sorrentino, C. Quintero-Quiroz, A. Aragoneses, M. C. Torrent, and C. Masoller, Opt. Express **23**, 5571 (2015).

[12] S. Barland et al., IEEE J. Quantum Electron. **41**, 1235 (2005).
